# Efficacy of Radial Pressure Wave Therapy Combined With Exercise in Physically Active Athletes With Patellar Tendinopathy: A Randomized Controlled Trial

**DOI:** 10.1155/tsm2/5598982

**Published:** 2026-07-29

**Authors:** Ishin Togashi, Masashi Nagao, Hirofumi Nishio, Keiji Kobayashi, Yohei Kobayashi, Jun Shiozawa, Yuki Someya, Yuki Shiota, Takayuki Komatsu, Hiroshi Ikeda, Muneaki Ishijima, Yuji Takazawa

**Affiliations:** ^1^ Department of Sports Medicine and Sportology, Juntendo University Graduate School of Medicine, 2-1-1 Hongo Bunkyo-ku, Tokyo 113-8421, Japan, juntendo.ac.jp; ^2^ Department of Sports Medicine, Juntendo University, Hongo, Tokyo, Japan, juntendo.ac.jp; ^3^ Innovative Medical Technology Research & Development Center, Juntendo University, Hongo, Tokyo, Japan, juntendo.ac.jp; ^4^ Department of Orthopaedics, Faculty of Medicine, Juntendo University, Hongo, Tokyo, Japan, juntendo.ac.jp; ^5^ Department of Medicine for Orthopedics and Motor Organ, Juntendo University Graduate School of Medicine, Hongo, Tokyo, Japan, juntendo.ac.jp; ^6^ J Medical Oyumino, Oyumino, Chiba, Japan; ^7^ Department of Physical Therapy, Faculty of Health Science, Juntendo University, Hongo, Tokyo, Japan, juntendo.ac.jp

**Keywords:** patellar tendinopathy, radial pressure wave, VISA-P-J

## Abstract

**Background:**

This study aimed to compare the effects of radial pressure wave (RPW) combined with exercise and exercise alone in athletes with patellar tendinopathy.

**Methods:**

This open‐label randomized controlled trial was conducted at the Juntendo University Sakura Campus Athletic Training Room in Chiba, Japan. Thirty‐three athletes with chronic patellar tendon pain due to patellar tendinopathy (mean age ± SD: 20.3 ± 2.9 years) were randomly allocated to either the RPW (*n* = 17) or the control (*n* = 16) group. The RPW group received four weekly treatment sessions over a four‐week period. Both groups were instructed by a physiotherapist and performed daily lower extremity stretching and strengthening exercises. The Japanese version of the Victorian Institute of Sports Assessment for Patellar Tendinopathy (VISA‐P‐J) was assessed at baseline and at 4, 16, and 28 weeks after the intervention.

**Results:**

The mean baseline VISA‐P‐J scores (95% confidence interval) were 68.8 (61.7–75.9) for the RPW group and 67.4 (60.1–74.8) for the control group. At 4, 16, and 28 weeks after the intervention, the RPW group obtained scores of 77.6 (70.4–84.9), 82.5 (74.8–90.2), and 89.9 (82.5–97.3), whereas the control group obtained scores of 72.7 (65.2–80.1), 78.5 (70.6–86.4), and 77.4 (69.8–85.1), respectively. Both groups showed significant improvements over time. Although the RPW group demonstrated greater improvements at 28 weeks in unadjusted analyses, these differences lost significance following adjustment for symptom duration.

**Conclusions:**

Both groups achieved clinically meaningful pain relief over the follow‐up period. Although participants in the RPW group reached the MCID earlier than those in the control group, no statistically significant between‐group differences were observed after adjustment for symptom duration.

**Trial Registration:** The University hospital Medical Information Network (UMIN) Clinical Trials Registry (UMIN000030707).

## 1. Introduction

Patellar tendinopathy is a sports‐related condition frequently associated with activities involving repetitive jumping and turning movements [1, 2]. Its primary clinical symptom is pain, which arises from the loading of the knee extensors [3]. Histopathologically, patellar tendinopathy is characterized by structural degeneration of the tendon tissue [4]. Degenerative changes are typically caused by the overloading of normal tendons, progressing through stages described in the continuum model [5]. Reactive tendinopathy is characterized by a noninflammatory proliferative response of the tendon to acute overload without structural disruption. Tendon disrepair represents a failed healing response with increased matrix disorganization and vascularity. Degenerative tendinopathy is characterized by extensive cell death, disorganized collagen matrix, and areas of necrosis, typically seen in chronically overloaded tendons. A cross‐sectional study of 100 athletes with patellar tendinopathy managed conservatively or surgically reported that approximately 33% were unable to return to sports activities within 6 months, significantly affecting their athletic careers. In addition, many athletes continue to compete while experiencing persistent pain [6]. Exercise interventions using eccentric contractions have been demonstrated to be effective and are widely regarded as the standard of care in clinical practice [7]. Consequently, numerous studies have focused on alleviating pain caused by patellar tendinopathy through exercise interventions and physical therapy.

Extracorporeal shock wave therapy (ESWT) is commonly employed in clinical practice to promote pain relief and tissue regeneration. It is anticipated to have a synergistic effect when combined with exercise interventions [8]. Radial pressure wave (RPW) therapy is a subtype of ESWT based on the ballistic generation principle, in which a projectile accelerated by compressed air collides with an applicator, generating a pressure wave that propagates radially from the applicator surface with intensity decreasing with tissue depth [9]. Meta‐analyses have demonstrated the efficacy of RPW in improving pain and restoring function [10, 11]. Furthermore, RPW intervention has been reported to have minimal adverse effects in the management of tendinopathy, including patellar tendinopathy, establishing it as a generally safe treatment option. Although the efficacy of ESWT for tendinopathies has been extensively studied [10, 12–14], only a limited number of investigations have specifically focused on patellar tendinopathy [10]. Moreover, RPW is rarely used alone in clinical practice and is typically combined with physical therapy. However, most studies have examined the efficacy of RPW compared to interventions that do not incorporate exercise [13, 14]. We hypothesized that the combination of RPW and exercise interventions has additional benefits over exercise interventions alone. This study thus aimed to compare the effects of RPW and exercise interventions with those of exercise interventions alone in patients with patellar tendinopathy.

## 2. Methods

### 2.1. Study Design

In this open‐label, randomized controlled trial, participants were randomly assigned to the RPW group or control group. This study was conducted in the Athletic training room at the Juntendo University Sakura Campus from October 2020 to February 2023. The RPW group underwent four RPW sessions at 1‐week intervals using a Radial Shockwave Physio Shockmaster (Sakai Medical Co., Ltd., Tokyo, Japan), with energy levels controlled to the participant’s “maximum comfortable tolerance” [15]. The RPW and control groups were instructed to perform home‐based exercise daily and attended weekly follow‐up clinic visits for exercise monitoring until 4 weeks after the study commenced. The follow‐up period lasted 24 weeks, and patient‐reported outcome measures and ultrasound‐based assessment of the degree of degenerative change in the tendon were collected at baseline, 4 (postintervention), 16 (12 weeks postintervention), and 28 weeks (24 weeks postintervention). The sample size was estimated using G∗Power software (latest Version 3.1.9.7; Heinrich Heine Universität Düsseldorf, Düsseldorf, Germany). The sample size was calculated based on the Japanese version of the Victorian Institute of Sports Assessment for Patellar Tendinopathy (VISA‐P‐J) score as the primary outcome for the between‐group comparison at 28 weeks, using a repeated measures ANOVA model with an assumed effect size of *f* = 0.25, 80% power, and a two‐sided *α* level of 0.05, yielding a minimum of 12 participants per group. The participants were randomly assigned to each group using REDCap, with sex and disease severity [16] used as allocation factors. Allocation sequences were generated by REDCap prior to enrollment, ensuring concealment of group assignment until eligibility was confirmed. Enrollment of eligible participants and assignment to interventions were both conducted by IT. As this was an open‐label trial, outcome assessments were also conducted by IT, who was aware of group allocation, and assessor blinding was not implemented.

### 2.2. Participants

Participants with chronic patellar tendon pain due to patellar tendinopathy who visited the Juntendo University Sakura Campus Athletic Training Room were enrolled. Athletes who (1) experienced patellar tendon pain for at least 1 month, (2) were aged at least 18 years at the time of consent, and (3) voluntarily provided consent after receiving a full explanation of the study were included in the analysis. Meanwhile, those who (1) had contraindications and precautions related to shock wave therapy, (2) who had a history of knee surgery, and (3) who had other trauma or injury to the knee joint during the study period were excluded. All participants continued to engage in their respective sports throughout the study period, despite experiencing patellar tendon pain.

### 2.3. Interventions

The RPW intervention was performed by a physiotherapist. The device was placed over the area experiencing the most pain. The RPW group underwent four sessions of RPW once a week for 4 weeks. Each session comprised 2000 shots at a frequency of 10 Hz, applied using a diameter 15‐mm stainless applicator (R15). The intensity of the pressure waves, measured in bars, was adjusted by the physiotherapist to the maximum comfortable level, which varied for each participant and was changed between sessions. In the present study, the mean pressure wave intensity values for the RPW group were 1.9 ± 0.3, 1.9 ± 0.2, 1.9 ± 0.2, and 2.0 ± 0.2 bar (mean ± standard deviation [SD]) for the first, second, third, and fourth intervention sessions, respectively. A pressure of 1.9–2.0 bar corresponds to an energy flux density of 0.07–0.15 mJ/mm^2^. During treatment, transmission gel was applied between the applicator and the participant’s skin. The exercise interventions comprised lower extremity stretching and strength training targeting the quadriceps and hamstring muscles [17]. Stretching exercises were performed for three sets of 10 s for each muscle group. Strength training also comprised three sets of 10 repetitions and primarily involved concentric and eccentric muscle contractions. The physiotherapists provided the participants with a booklet to guide them in completing the exercise interventions (Supporting Information 1). The exercise interventions were supervised by physiotherapists at each session. The exercise content was identical between the groups. Although adherence was not formally quantified, exercise sessions were supervised weekly, and physiotherapists confirmed verbal compliance at each visit. All participants attended 100% of the weekly supervised follow‐up sessions during the 4‐week intervention period.

### 2.4. Outcome Measures

The primary outcome measure was the change in the scores of the VISA‐P‐J. This scale quantifies pain and activity levels and is specifically designed to assess the outcomes of patients with patellar tendinopathy. Its validity and reliability have been confirmed in a previous study [18]. The VISA‐P‐J scores ranged from 0 to 100 (0 = no activity/maximum pain and 100 = maximum activity/no pain). The secondary outcome measures were changes in pain levels during sports, assessed using a visual analog scale (VAS), with scores ranging from 0 to 100 mm (0 mm = no pain and 100 mm = worst pain). The degree of degenerative changes in the tendon was assessed using a continuum model, which classified the conditions into reactive tendinopathy, tendon disrepair, and degenerative tendinopathy [5]. Tendon degeneration was classified using B‐mode ultrasonography (Applio 300, Canon, Tochigi, Japan) according to the continuum model [5]. All assessments were conducted by a physiotherapist.

### 2.5. Ethical Considerations

This study was approved by the Ethics Committee for Human Experiments of Juntendo University (no. 2020‐60). All participants were fully informed of the nature and rationale of the study. Written informed consent was obtained from all participants. The reporting of this randomized controlled trial conforms to the CONSORT guidelines.

### 2.6. Statistical Methods

Participant demographics were presented using descriptive statistics (mean and SD, numbers, and percentages). The normality of data distribution was assessed using the Shapiro–Wilk test. The Mann–Whitney *U* test was used to assess the differences in the baseline characteristics between the groups. The primary analysis was the between‐group comparison of VISA‐P‐J scores at 28 weeks, for which the study was powered. Between‐group comparisons at 4 and 16 weeks were considered secondary analyses. Repeated measures analysis of variance was applied to assess the differences in VISA‐P‐J and VAS scores between the groups over time, followed by the Bonferroni post hoc test. Between‐group differences with 95% confidence intervals were calculated at each follow‐up time point. In addition, although adjustment for symptom duration was not prespecified in the original study protocol, a post hoc exploratory analysis using analysis of covariance (ANCOVA) was conducted given the clinically meaningful difference in symptom duration between groups at baseline (RPW: 14.1 ± 16.2 weeks; control: 32.2 ± 38.1 weeks). This adjusted analysis is presented as the primary basis for interpreting between‐group differences, with unadjusted analyses reported for reference. The Friedman test was applied to assess the degree of degenerative change in the tendon. All relevant analyses were performed following the intention‐to‐treat principle. Missing outcome data were handled using the last observation carried forward (LOCF) method, as used in previous clinical trials [13]. A two‐sided *p* value of < 0.05 was considered significant. All statistical analyses were performed using the IBM SPSS Statistical software package Version 29 (SPSS Inc., Chicago, Illinois, USA).

## 3. Results

### 3.1. Study Participants

**FIGURE 1:**
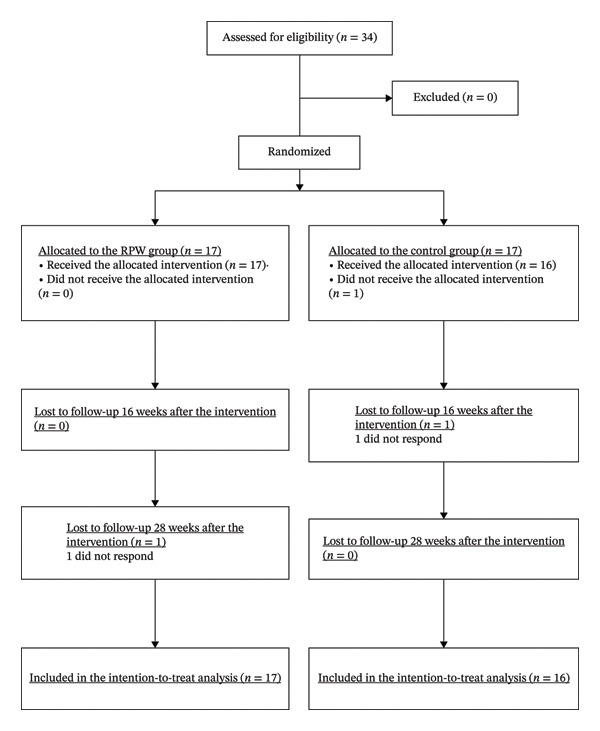
Flow diagram of the participant selection process. RPW: radial pressure wave.

Thirty‐four athletes were assigned to either the RPW or control groups. During the study, one athlete (5.9%) in the control group withdrew consent, whereas one athlete (5.9%) in the RPW group and one athlete (5.9%) in the control group were lost to follow‐up. Therefore, the final numbers of participants were 17 and 16 in the RPW and control groups, respectively (Figure 1). The baseline demographic characteristics of the participants are presented in Table 1.

**TABLE 1:** Characteristics of the participants (*n* = 33).

	RPW (*n* = 17)	Control (*n* = 16)	*p*	Total (*n* = 33)
Age (y)	20.1 ± 1.2	20.7 ± 4.0	0.444	20.3 ± 2.9

Gender (male), *n* (%)	11 (64.7)	10 (64.7)	0.929	21 (63.6)

Height (cm)	171.5 ± 10.6	170.9 ± 10.3	0.763	171.2 ± 10.3

Weight (kg)	72.0 ± 18.9	65.5 ± 10.8	0.817	68.8 ± 15.6

BMI	24.4 ± 5.2	22.3 ± 1.7	0.382	23.4 ± 4.0

Symptom duration (weeks)	14.1 ± 16.2	32.2 ± 38.1	0.157	22.9 ± 29.9

Primary sport	8 athletics	5 athletics		
	2 soccer	4 soccer		
	1 triathlon event	2 volleyball		
	1 swimming	2 baseball		
	1 volleyball	1 handball		
	1 badminton	1 ice hockey		
	1 lifesaving	1 inline alpine		
	1 futsal			
	1 squash			

*Note:* Values are expressed as the mean ± standard deviation.

### 3.2. VISA‐P‐J Scores

**FIGURE 2:**
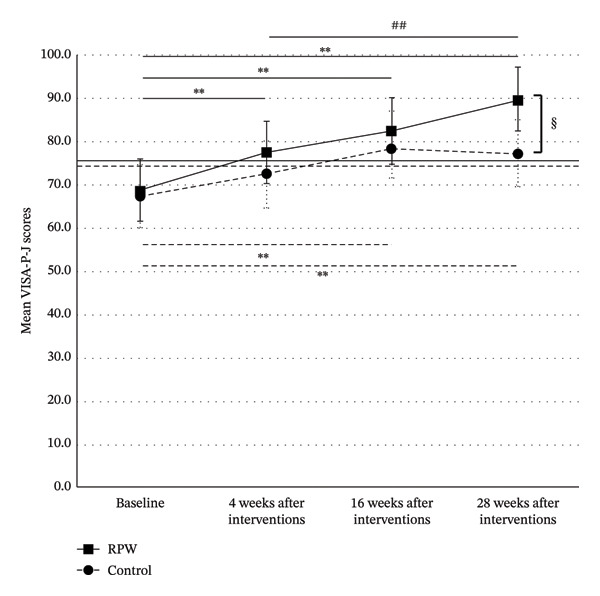
Mean VISA‐P‐J scores with 95% confidence intervals over 28 weeks. ^∗∗^ *p* < 0.01 (vs. baseline). ^##^ *p* < 0.01 (vs. 4 weeks after interventions). ^§^ *p* < 0.05 (vs. control). The horizontal line indicates the minimal clinically important difference threshold of 7 points from baseline for the VISA‐P‐J score. VISA‐P‐J: Victorian Institute of Sports Assessment for patellar tendinopathy (Japanese version); CI: confidence interval; RPW: radial pressure wave.

The mean baseline VISA‐P‐J scores (95% confidence interval) were 68.8 (61.7–75.9) for the RPW group and 67.4 (60.1–74.8) for the control group. The VISA‐P‐J scores of the RPW and control group were 77.6 (70.4–84.9) and 72.7 (65.2–80.1) at 4 weeks, 82.5 (74.8–90.2) and 78.5 (70.6–86.4) at 16 weeks, and 89.9 (82.5–97.3) and 77.4 (69.8–85.1) at 28 weeks, respectively. The mean changes in the VISA‐P‐J scores from baseline are shown in Figure 2. Compared to the baseline, the VISA‐P‐J scores in the RPW group significantly improved at 4 (*p* = 0.006), 16 (*p* = 0.003), and 28 weeks (*p* = 0.000009). In addition, the VISA‐P‐J scores in the RPW group significantly improved from 4 to 28 weeks (*p* = 0.003). Similarly, the VISA‐P‐J scores in the control group significantly improved at 16 and 28 weeks compared to baseline (*p* = 0.032 and *p* = 0.009, respectively). At 28 weeks, the RPW group showed a significant improvement in the VISA‐P‐J scores compared to the control group (*p* = 0.024) (Figure 2). Conversely, ANCOVA with adjustment for symptom duration showed no significant between‐group differences at baseline (*p* = 0.722), 4 weeks (*p* = 0.341), 16 weeks (*p* = 0.718), or 28 weeks (*p* = 0.063). The between‐group differences in VISA‐P‐J scores (RPW minus control), estimated from the repeated measures analysis, were 5.0 points (95% CI: −5.4–15.3) at 4 weeks, 4.0 points (95% CI: −7.0–15.1) at 16 weeks, and 12.4 points (95% CI: 1.8–23.1) at 28 weeks.

### 3.3. VAS Scores

**FIGURE 3:**
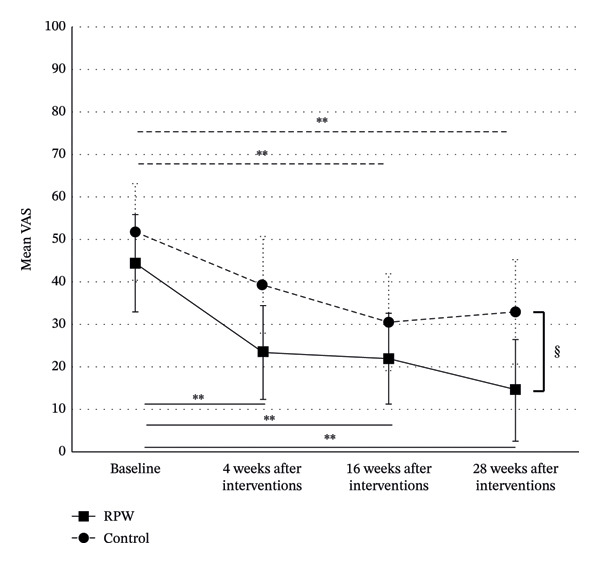
Mean VAS scores with 95% confidence intervals over 28 weeks. ^∗∗^ *p* < 0.01 (vs. baseline). ^§^ *p* < 0.05 (vs. control). VAS: visual analog scale; CI: confidence interval; RPW: radial pressure wave.

Compared to the baseline, the VAS scores in the RPW group significantly improved at 4 (*p* = 0.001), 16 (*p* = 0.001), and 28 weeks (*p* = 0.0002). Similarly, the VAS scores in the control group significantly improved compared to the baseline at 16 weeks (*p* = 0.004) and 28 weeks (*p* = 0.0041). At 28 weeks, the RPW group showed a significant improvement in VAS scores compared to the control group (*p* = 0.038) (Figure 3). Conversely, ANCOVA with adjustment for symptom duration showed no significant between‐group differences at baseline (*p* = 0.469), 4 weeks (*p* = 0.06), 16 weeks (*p* = 0.328), or 28 weeks (*p* = 0.162). The between‐group differences in VAS scores (RPW minus control) were −15.7 points (95% CI: −31.9–0.5) at 4 weeks, −8.6 points (95% CI: −24.4–7.2) at 16 weeks, and −18.2 points (95% CI: −35.4–1.0) at 28 weeks.

### 3.4. Degree of Degenerative Change in the Tendon

Regarding the degree of degenerative change in the tendons, no significant within‐group changes were observed in either the RPW (*p* = 0.106) or control (*p* = 0.112) groups (Table 2).

**TABLE 2:** The degree of degenerative change in the tendon at baseline, 4 weeks after intervention, 16 weeks after intervention, and 28 weeks after intervention in the RPW and control groups.

	RPW group (*n* = 17)				Control group (*n* = 16)			
	Baseline	4 weeks after the interventions	16 weeks after the interventions	28 weeks after the interventions	Baseline	4 weeks after the interventions	16 weeks after the interventions	28 weeks after the interventions
Reactive tendinopathy, *n* (%)	11 (64.7)	15 (88.2)	13 (76.5)	14 (82.4)	11 (68.8)	11 (68.8)	12 (75.0)	12 (75.0)
Tendon disrepair, *n* (%)	5 (29.4)	2 (11.8)	4 (23.5)	2 (11.8)	4 (25.0)	5 (31.0)	4 (25.0)	4 (25.0)
Degenerative tendinopathy, *n* (%)	1 (5.9)	0 (0)	0 (0)	1 (5.9)	1 (6.3)	0 (0)	0 (0)	0 (0)

Abbreviation: RPW, radial pressure wave.

## 4. Discussion

Both groups showed significant improvements in the VISA‐P‐J and VAS scores compared to the baseline. Overall, the RPW group demonstrated numerically greater improvements at 28 weeks in the unadjusted analyses. However, these differences were attenuated following adjustment for symptom duration and were no longer statistically significant. Therefore, no definitive additional treatment effect of RPW over exercise alone was demonstrated in the present study. No adverse events were observed during the study period.

The minimal clinically important difference (MCID) represents the smallest difference in an outcome measure between pre‐ and postintervention that is perceived as beneficial or detrimental by the patient [19]. A previous study identified that the MCID of the VISA‐P‐J score for patellar tendinopathy was 7 points [18]. In the present study, the RPW group achieved a 7‐point improvement in VISA‐P‐J scores at 4 weeks, whereas the control group only achieved the same improvement in the VISA‐P‐J scores at 16 weeks. This demonstrates that the combination of RPW and exercise interventions led to a more rapid achievement of the MCID of the VISA‐P‐J scores compared to exercise interventions alone. This finding is consistent with those of van der Worp et al., who reported significant within‐group improvements in VISA‐P scores following both focused and radial shockwave therapy in patients with patellar tendinopathy, with no significant difference between treatment modalities at any time point [10]. In this study, the VISA‐P score improved by more than 7 points at 4 weeks following the intervention [10]. In addition, the improvement was sustained for at least 28 weeks postintervention. These findings suggest earlier clinically meaningful improvement with RPW combined with exercise, although this interpretation should be viewed cautiously given the lack of significant between‐group differences after adjustment.

Although RPW intervention has been reported to alleviate pain, its underlying mechanism remains unknown [8, 20]. Although no definitive conclusion has been reached [11], tissue regeneration is considered a potential explanation for pain relief [8]. A review of best practices for ESWT in musculoskeletal medicine proposed several mechanisms of action relevant to tendinopathy, including the promotion of angiogenesis, modulation of inflammatory mediators, stimulation of tenocyte proliferation, and inhibition of nociceptive nerve pathways [15]. These proposed mechanisms are consistent with the pain relief observed in both groups in the present study; however, which of these mechanisms predominantly contribute to the clinical effects of RPW in patellar tendinopathy remains to be clarified. Previous studies investigating the impact of RPW have primarily focused on VISA‐P scores and pain as outcomes [10, 21], leaving uncertainty on whether tendon tissue regeneration occurs. A review reported that disease severity, assessed through ultrasound imaging, does not always correlate with pain symptoms [22]. In fact, our results were consistent with those of a previous study, which showed that the degree of degenerative change in the tendon did not change throughout the study period [10]. Another potential mechanism involves the sprouting of free nerve endings. Free nerve endings contain pain‐sensing receptors, and their sprouting is considered a potential cause of pain in patients with patellar tendinopathy [23]. One previous study showed that RPW application resulted in a reduction in the Protein gene product 9.5 and calcitonin gene–related peptide levels, both of which serve as markers of free nerve endings [20]. Therefore, the pain relief observed with RPW may be due to the degeneration of free nerve endings. Although the protein expression levels were not assessed, this potential mechanism warrants further investigation.

In one previous study comparing RPW alone with a placebo, no significant efficacy was observed for RPW alone, as both groups demonstrated similar changes within 22 weeks [13]. By contrast, another study comparing RPW with other conservative interventions demonstrated that the RPW intervention was superior to the physiotherapy intervention [14]. In the present study, both groups demonstrated significant within‐group improvements; however, no statistically significant between‐group difference was observed after adjustment for symptom duration. As this study does not include a placebo group, we are unable to conclude that RPW alone is effective over placebo. We hypothesize that the pain‐relieving effects of RPW enhanced the effectiveness of the exercise intervention, resulting in synergistic improvement. Although the effects of the interventions plateaued as the VISA‐P score approached 80 points in both groups, the RPW group demonstrated greater improvements during the later stage. This result may be more evident in participants with a shorter symptom duration. It is plausible that RPW therapy may be particularly effective in athletes with less chronic tendinopathy, as earlier‐stage disease may retain greater tissue reactivity and capacity for repair. Future studies prospectively stratifying participants by symptom duration are warranted to investigate this hypothesis.

This study has some limitations. First, the participants were recruited from a single institution, which limited the demographic diversity of the sample. The study included university students actively involved in competitive sports clubs. Therefore, the findings may be applicable to competitive‐level athletes but not to individuals participating in recreational sports. Second, detailed information regarding the amount and quality of the exercise performed during the study was not available. In the present study, a standardized exercise protocol was implemented; however, pain tolerance thresholds during exercise were not formally specified, and co‐interventions determined by the treating physiotherapists were not systematically recorded. Although the physiotherapists consistently aligned the exercise content with the shared objective of improving lower extremity function in patients with patellar tendinopathy, variability in the exercise interventions cannot be fully excluded. In addition, home‐based exercise adherence was confirmed only verbally during weekly supervised sessions and was not formally quantified, which limits the objective assessment of compliance with the home‐based exercise program. Third, the mean RPW intensity applied in the present study (1.9–2.0 bar) was at or slightly below the lower boundary of the ISMST‐recommended range of 2–4 bar for patellar tendinopathy [9]. Although intensity was adjusted to each participant’s maximum comfortable tolerance, this may represent a relative undertreatment and could have contributed to the lack of statistically significant between‐group differences in the adjusted analyses. Fourth, missing outcome data were handled using LOCF, which assumes no change after dropout and may introduce bias in longitudinal estimates. Although only two participants had missing data, this limits the expected impact of the imputation method on the study findings. Fifth, all outcome assessments were conducted by IT, the same individual who enrolled and assigned participants to interventions, without blinding to group allocation. Given that the primary and secondary outcomes are patient‐reported outcome measures, the risk of ascertainment and expectation bias is nontrivial. The direction of this potential bias would likely favor the RPW group, as participants in the RPW group may have reported greater perceived improvement relative to those in the control group.

## 5. Conclusions

Both groups achieved clinically meaningful pain relief over the follow‐up period. Although participants in the RPW group reached the MCID earlier than those in the control group, no statistically significant between‐group differences were observed after adjustment for symptom duration.

**Nomenclature**
ESWT: Extracorporeal shock wave therapy
RPW: Radial pressure wave
VISA‐P‐J: Japanese version of the Victorian Institute of Sports Assessment for Patellar Tendinopathy
VAS: Visual analog scale
ANCOVA: Analysis of covariance
LOCF: Last observation carried forward
MCID: Minimal clinically important difference

## Funding

This study was funded by Sakai Medical Co., Ltd.

## Disclosure

This study was conducted in cooperation with Sakai Medical Co., Ltd. The sponsor had no role in the study design, data collection, statistical analysis, interpretation of results, manuscript preparation, or decision to submit for publication.

## Ethics Statement

The study was conducted in accordance with the 1964 Declaration of Helsinki and its later amendments or comparable ethical standards. The study protocol was approved by the Ethics Committee of the Juntendo University, Japan (no. 2020‐60). Written informed consent to participate was obtained from all participants. The study was registered in the UMIN Clinical Trials Registry.

## Consent

Please see the Ethics Statement.

## Conflicts of Interest

The authors declare no conflicts of interest.

## Supporting Information

Additional supporting information can be found online in the Supporting Information section.

## Supporting information


**Supporting Information** Exercise booklet for patellar tendinopathy.

## Data Availability

The data that support the findings of this study are available from the corresponding author upon reasonable request.
