# Supplementary material for: Efficacy of Radial Pressure Wave Therapy Combined With Exercise in Physically Active Athletes With Patellar Tendinopathy: A Randomized Controlled Trial
Source: Transl Sports Med. 2026 Jul 29;2026:5598982. doi: 10.1155/tsm2/5598982 (PMC13416398; doi:10.1155/tsm2/5598982)

Supplementary Information 1: Exercise booklet for patellar tendinopathy

This supplementary file provides the exercise booklet used in the study presented in the main manuscript entitled "Efficacy of radial pressure wave therapy combined with exercise in physically active athletes with patellar tendinopathy: A randomized controlled trial."

A booklet to guide them in completing the exercise interventions (original Japanese version)


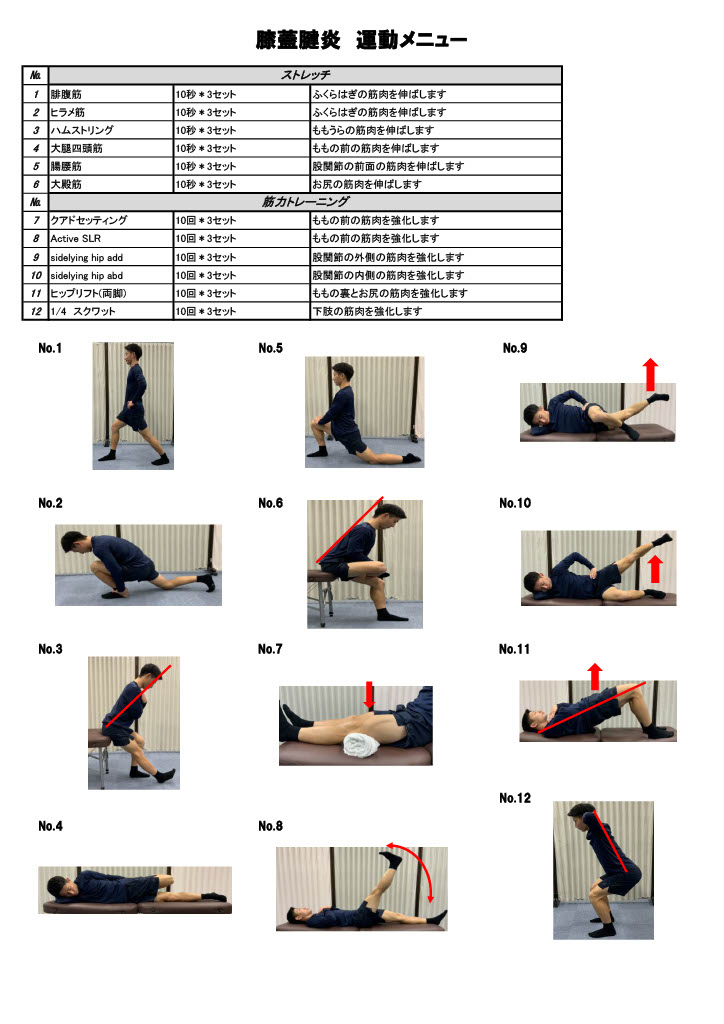


A booklet to guide them in completing the exercise interventions (English translation version)


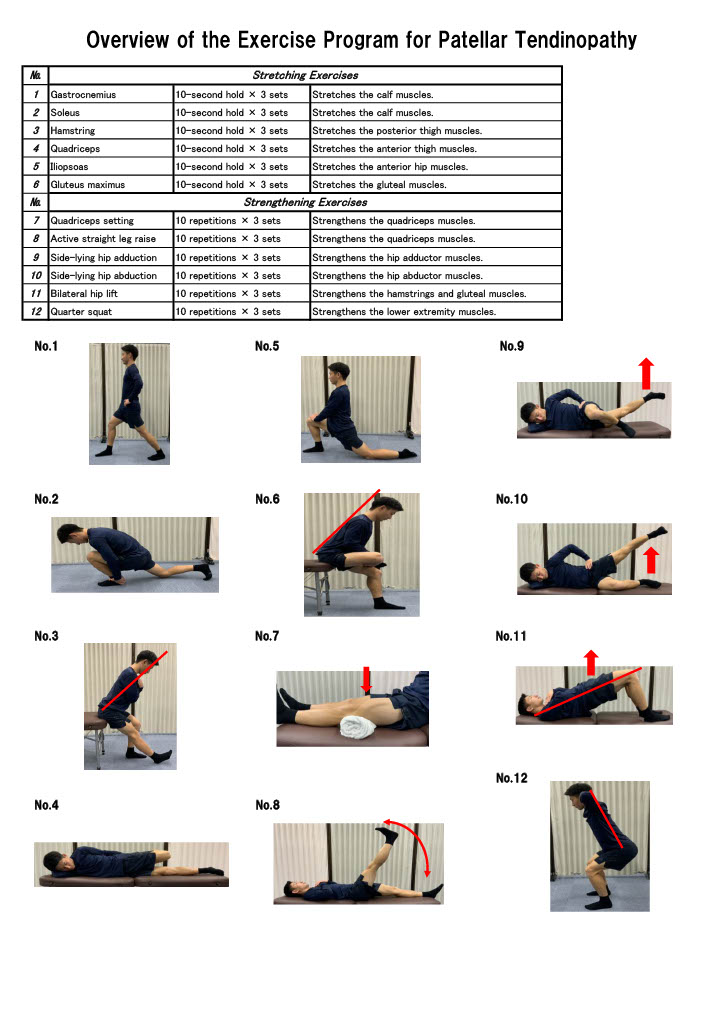

Supplement: Supplementary file 1 — Supporting Information Exercise booklet for patellar tendinopathy. [file TSM2-2026-5598982-s001.docx]
